# Adaptation to the Direction of Others’ Gaze: A Review

**DOI:** 10.3389/fpsyg.2018.02165

**Published:** 2018-11-09

**Authors:** Colin W. G. Clifford, Colin J. Palmer

**Affiliations:** School of Psychology, University of New South Wales Sydney, Sydney, NSW, Australia

**Keywords:** gaze direction, visual adaptation, social attention, face perception, sensory coding

## Abstract

The direction of another person’s gaze provides us with a strong cue to their intentions and future actions, and, correspondingly, the human visual system has evolved to extract information about others’ gaze from the sensory stream. The perception of gaze is a remarkably plastic process: adaptation to a particular direction of gaze over a matter of seconds or minutes can cause marked aftereffects in our sense of where other people are looking. In this review, we first discuss the measurement, specificity, and neural correlates of gaze aftereffects. We then examine how studies that have explored the perceptual and neural determinants of gaze aftereffects have provided key insights into the nature of how other people’s gaze direction is represented within the visual hierarchy. This includes the level of perceptual representation of gaze direction (e.g., relating to integrated vs. local facial features) and the interaction of this system with higher-level social-cognitive functions, such as theory of mind. Moreover, computational modeling of data from behavioral studies of gaze adaptation allows us to make inferences about the functional principles that govern the neural encoding of gaze direction. This in turn provides a foundation for testing computational theories of neuropsychiatric conditions in which gaze processing is compromised, such as autism.

## Introduction

Eye gaze signals play a critical role in human communication and interaction (Argyle and Cook, 1976). To an observer, the direction of your gaze reveals where you are looking and hence what you are looking at. This might be an object of shared attention or it might be the observer him or herself. The direction of your gaze is thus a strong social signal to your intentions and future actions (Baron-Cohen, 1995), and gaze plays a role in many social behaviors that rely on interpersonal coordination of attention and behavior, such as learning, and joint action (Frith and Frith, 2008). Understanding the mechanisms by which another’s gaze is perceived and interpreted has consequently become an active area of interest in the burgeoning field of social neuroscience (Nummenmaa and Calder, 2009).

The perception of gaze direction is an interesting phenomenon to study in part because it sits at the interface between visual perception and social cognition. Psychophysics and neuroimaging research has begun to reveal how the human visual system extracts information about another person’s focus of attention from the stream of sensory signals that are relayed from the retina to the cortex (Langton, 2010), the role played by sub-cortical structures such as the superior colliculus, amygdala and pulvinar (e.g., in signaling eye contact; Senju and Johnson, 2009), and the interaction of these systems with higher-level attentional and cognitive processes (Carlin and Calder, 2013). Gaze direction is a component of our social experience that is relatively tractable for experimental research, as it can be defined along a continuous dimension (e.g., with horizontal deviations of the eyes ranging from approximately 40° leftward to 40° rightward) and has an identified cortical basis in the superior temporal sulcus (STS, Carlin and Calder, 2013). In this way, perception of gaze direction may serve as an important model system for social neuroscience.

The phenomenon of *visual adaptation* to gaze direction demonstrates that our perception of other people’s gaze is a remarkably plastic process that can be affected by the recent history of stimulation (Jenkins et al., 2006; Seyama and Nagayama, 2006). Adaptation is an overloaded term that refers to three inter-related elements: procedure, process and percept (Wade and Verstraten, 2005). The procedure of adaptation is exposure to a particular diet of sensory stimulation. In response to changes in stimulation, our sensory systems change the way that they process incoming information. These changes in sensory processing give rise to measurable aftereffects in our perception. Adaptation is well-established as a fundamental characteristic of low-level sensory processing, readily apparent for sensory properties like luminance, orientation, and color (Webster, 2015). It is only relatively recently, however, that adaptation to higher-level visual qualities associated with faces and objects has been explored (Clifford and Rhodes, 2005). In the context of eye gaze, an observer who has been adapted to a series of faces displaying averted gaze will tend to display marked changes in their perception of others’ gaze direction, such as whether they judge a given face stimulus as looking at them or not (Jenkins et al., 2006; Seyama and Nagayama, 2006). Investigation of the details of this phenomenon has provided important insights into how the direction of another person’s gaze is coded in the visual system.

Here, we review the literature on gaze adaptation and discuss its implications for our understanding of gaze processing in the human brain. The following section focuses on establishing the nature of gaze aftereffects. We begin with methods to measure the effects of adaptation on gaze perception, both in terms of perceptual effects and their neural correlates. These measures allow us to ask to what extent the effects of adaptation are specific to eye gaze, and at what level(s) of the visual processing hierarchy they are mediated. Importantly, the determinants and phenomenology of adaptation are also diagnostic as to the processes by which our brains represent the direction of the gaze of others. Correspondingly, Section “What Gaze Aftereffects Reveal About the Sensory Coding of Gaze Direction” focuses on how gaze aftereffects can be used experimentally to probe the sensory coding of gaze direction in the brain. The properties of gaze adaptation allow us to characterize the representation of gaze direction in the neurotypical human visual system in terms of a simple channel structure, and identify functional mechanisms that the sensory coding of gaze direction may rest upon. This, in turn, allows us to test theoretically motivated hypotheses about gaze processing in clinical populations such as people with autism spectrum disorder (ASD).

## The Nature of Gaze Aftereffects

### Measurement of Gaze Aftereffects

Gaze aftereffects are generally ‘repulsive’ or ‘negative.’ That is to say, following adaptation to a series of faces with gaze averted in a particular direction, the perceived gaze direction of a subsequently presented face is *repelled away* from this adapting direction when compared to how that same face was perceived in an unadapted baseline condition. Robust gaze aftereffects are evident with various techniques of measurement. For example, using a forced-choice judgment of gaze as leftward or rightward, Seyama and Nagayama (2006) found that adaptation to gaze averted horizontally by 35° biased the perception of subsequently presented test faces throughout the range ± 4° such that they were more likely to be reported as gazing in the opposite direction to the adaptor. Similarly, using a forced-choice categorization of gaze as leftward/direct/rightward, Jenkins et al. (2006) found that adaptation to 25° averted gaze tended to cause test stimuli averted 5–10° to the same side as the adaptor to be reported as gazing directly at the observer.

**FIGURE 1:**
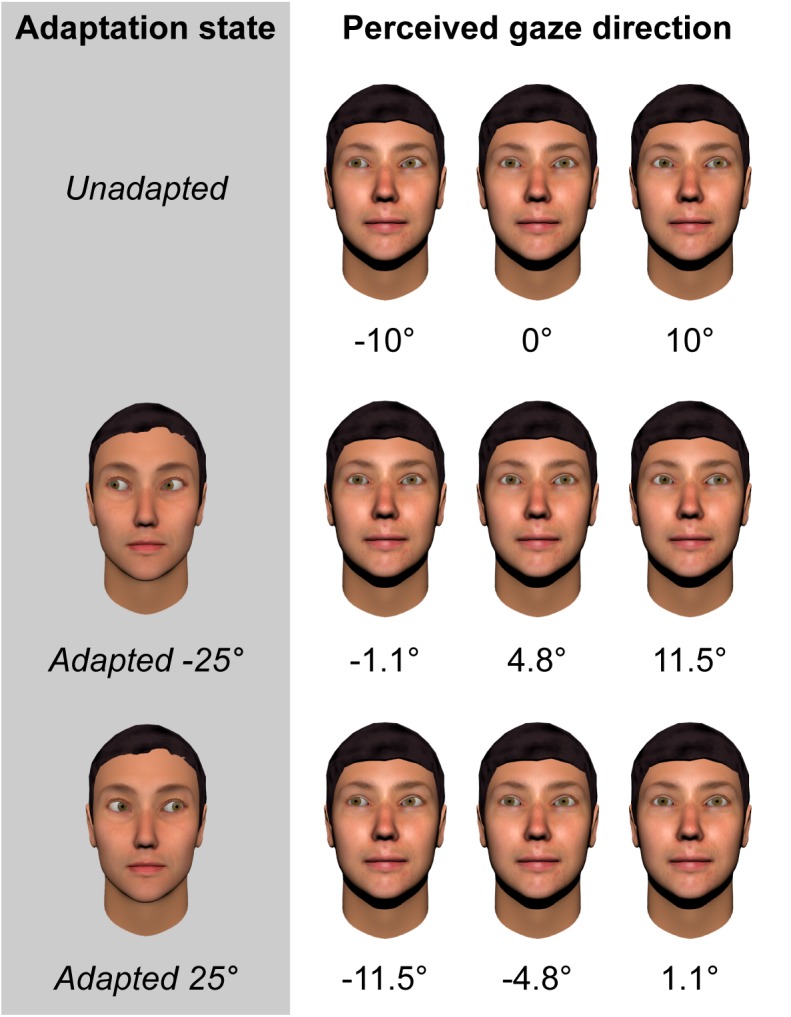
Illustration of the magnitude of gaze aftereffects based on fitted data from 28 neurotypical adults (Palmer et al., 2018). Leftmost column denotes the adapting condition. Subsequent columns represent the perceived direction of gaze of faces with eyes averted horizontally by –10, 0, and +10 degrees, respectively.

More recent studies have used a continuous rather than categorical measure of perceived gaze direction, requiring participants to adjust an on-screen pointer to indicate the direction that a face appears to be looking (Palmer and Clifford, 2017a,b; Palmer et al., 2018). Using a pointer has the advantage of allowing the effects of adaptation to be measured metrically (e.g., how much the perceived gaze direction of a given face shifts in degrees as a consequence of adaptation), and across the whole gamut of physically realizable gaze directions. These studies have consistently found that, for adaptors averted by 25°, the strongest aftereffects are observed for test stimuli averted by around 10° to the same side as the adaptor. The peak magnitude of these aftereffects is approximately 8°, which corresponds to roughly half the width of the participant’s head at the viewing distance of 50 cm used in these studies. Thus, adaptation caused gaze directed at the participant’s ear to appear to be directed straight at them! This is illustrated in Figure 1.

Gaze aftereffects can be surprisingly long lasting, surviving up to 24 h when there is no testing immediately after adaptation (Kloth and Rhodes, 2016; Kloth et al., 2017) but decaying with repeated testing (Kloth and Schweinberger, 2008; Kloth et al., 2017). Most studies of gaze adaptation have investigated gaze averted horizontally. However, Cheleski et al. (2013) demonstrated comparable degrees of adaptation to gaze averted vertically or obliquely. Adaptation has also been demonstrated to gaze *vergence*, the relative deviation of the eyes that indicates the depth at which someone is fixating (Stiel et al., 2014).

### Neural Correlates of Gaze Adaptation

The neural correlates of gaze adaptation have been investigated using both functional magnetic resonance imaging (fMRI: Calder et al., 2007) and electroencephalography (EEG: Schweinberger et al., 2007; Kloth and Schweinberger, 2010). Using fMRI, Calder et al. (2007) assessed how adaptation to gaze averted left or right by 25° affected the blood oxygenation level dependent (BOLD) response to test faces with gaze averted 10° left, 10° right or direct. They observed adaptation of the BOLD response in the anterior STS and inferior parietal lobule (IPL) specific to faces with gaze averted to the same side as the adaptor. These effects showed significant lateralization to the right hemisphere.

Schweinberger et al. (2007) used EEG to measure event-related potentials in response to gaze averted 5° left, 5° right or direct following adaptation to gaze averted left or right by 25°. They observed effects specific to gaze direction not in the N170 but in later (∼250–350 ms) occipitotemporal components. These findings were confirmed and extended by Kloth and Schweinberger (2010), who also observed direction-specific adaptation in a late (∼400–600 ms) positive centroparietal component.

Together, these studies demonstrate the sensitivity of adaptation as a technique to dissociate the neural systems coding different directions of gaze. Future studies combining the relatively high spatial resolution of fMRI with the temporal precision of EEG or magnetoencephalography (MEG) could further our understanding of the mechanisms involved. Consistent with the effect of adaptation to gaze direction on the BOLD response in humans, single-unit recording studies in macaques have identified cells in the anterior STS that respond selectively to the gaze direction of observed faces (Perrett et al., 1985; De Souza et al., 2005). The neural correlates of gaze adaptation in humans thus fit with a picture of gaze-selective processing of faces in higher-order visual pathways in temporal cortex (Carlin and Calder, 2013). However, the effects of prolonged or repeated presentation of faces on the responses of gaze-sensitive cells is yet to be examined with extracellular recording techniques. In addition, sub-cortical structures have been implicated in gaze processing, such as the superior colliculus, amygdala, and pulvinar in signaling eye contact (Senju and Johnson, 2009), but whether gaze aftereffects reflect changes in processing within gaze-specific sub-cortical systems is yet to be determined.

### Specificity of Gaze Adaptation

A fundamental question regarding gaze adaptation is whether the effects are specific to the visual processing of eye gaze. It is helpful to decompose this question into two parts. Firstly, is adaptation occurring at a level at which the direction of gaze is represented, rather than being generated earlier in the visual processing hierarchy? If so, are the effects specific to eye gaze or do they generalize to other directional cues to social attention (e.g., pointing gestures)?

#### Is Gaze Adaptation ‘High-Level’?

It has been suggested that perceptual face aftereffects may in large part be generated by the adaptation of early visual mechanisms, such as those that represent local orientation (e.g., Dickinson and Badcock, 2013). While such early mechanisms are insensitive to faces *per se*, they provide the input to higher, face-selective levels of visual processing. Thus, adaptation of low-level mechanisms could change the input to those representations coding facial attributes explicitly. If the effects of adaptation on the representation of gaze direction were simply inherited from earlier levels of processing then this would undoubtedly make them rather less interesting theoretically, although of course no less compelling perceptually.

To reduce the effects of adaptation at low-level, retinotopic stages of processing on face aftereffects, studies typically introduce a size change between adapting and test stimuli such that corresponding regions no longer overlap spatially (e.g., Zhao and Chubb, 2001). When Jenkins et al. (2006) doubled the size of the adapting stimuli while leaving test size the same, this manipulation had very little effect on the pattern of gaze aftereffects they observed. Gaze adaptation has also been shown to survive changes in viewpoint (i.e., yaw rotation of the head) between adaptor and test, which provides an alternative means of disrupting low-level correspondences (Jenkins et al., 2006; Palmer and Clifford, 2017b).

However, the importance of controlling for the inheritance of low-level adaptation effects is illustrated by a study of gaze adaptation under inter-ocular suppression (Stein et al., 2012). Stein et al. (2012) used the technique of continuous flash suppression (Tsuchiya and Koch, 2005) to render their adapting gaze stimuli perceptually invisible. To achieve this, they presented the adaptors to only one eye while the corresponding region of retina in the other eye was stimulated with a stream of continuously changing, colorful patterns. They then presented fully visible test stimuli. When there was no size change between adaptor and test, Stein et al. (2012) found significant aftereffects of adaptation to the perceptually invisible stimuli, even when the test was presented only to the eye opposite to the adaptor. However, when a size difference of 25% was introduced between adaptor and test, gaze aftereffects to invisible stimuli were abolished. Thus, the study of Stein et al. (2012) not only demonstrates that awareness of the adapting stimulus is required for gaze-specific aftereffects to be generated, it highlights the importance of introducing a manipulation such as a size change between adaptor and test in order to avoid the effects of adaptation at lower levels of processing propagating up through the visual hierarchy.

One can also consider different levels of visual processing at which adaptation might occur *within* the domain of face perception. Our sense of where other people are looking depends on the *integration* of different facial features, namely integration of information about the two eyes (Nguyen et al., 2018) and integration of information about eye direction and head orientation (Wollaston, 1824; Otsuka et al., 2016). Thus, key to defining the nature of gaze aftereffects is whether adaptation acts on representations of where other people are looking that are derived from the integration of different facial cues.

First, Stiel et al. (2014) investigated whether gaze adaptation occurs at a level of representation within the visual hierarchy at which information about the deviations of the stimulus’s two eyes is integrated. They used two different adapting conditions in which each eye alternated in deviation between 20° left or right. In the ‘averted’ condition, each eye was always deviated in the same direction as the other such that gaze alternated between adapting faces in a series from left to right with eyes parallel. In the ‘vergent’ condition, the deviation of the two eyes was always opposite such that gaze alternated between adapting faces from converged (i.e., ‘cross-eyed’) to diverged. Crucially, the behavior of each eye, when considered independently, was the same in the two adapting conditions – alternating between leftward and rightward deviated with a period of 3 s. However, in the ‘averted’ condition the two eyes alternated in phase with one another (left, right, left, right …) whereas in the ‘vergent’ condition they alternated in anti-phase (converged, diverged, converged, diverged …). Because the deviation of each individual eye in each adaptation condition followed the same duty cycle, a difference between these two conditions in the resulting aftereffects could be attributed to adaptation of an *integrated* representation of the two eyes’ gaze, rather than adaptation to the features of either eye alone.

Stiel et al. (2014) compared the effects of these two adaptation conditions on both the perception of gaze direction and the perception of gaze vergence. They observed a greater increase in the range of test *gaze directions* categorized by observers as directed at them following ‘averted’ compared to ‘vergent’ adaptation. Conversely, the range of test *gaze vergences* categorized as parallel (as opposed to convergent or divergent) was greater for the ‘vergent’ than the ‘averted’ adapted condition. This specificity indicates that both adaptation to gaze direction and gaze vergence occur at a level of processing at which information from the two eyes is integrated.

Furthermore, the results of Stiel et al. (2014) demonstrated that adaptation occurs not only to the two eyes as a unitary stimulus but also to the individual eyes independently. In particular, comparison of the cross-adaptation conditions to an unadapted baseline revealed significant effects both of ‘vergent’ adaptation on perception of gaze direction and of ‘averted’ adaptation on vergence perception, even though there was a size difference between adaptor and test. Such two-way cross-adaptation is indicative of adaptation at a size-tolerant level of representation of the *individual* eyes that occurs prior to the integration of information from the two eyes to extract a unique direction and depth of fixation.

More recently, Palmer and Clifford (2018) investigated whether adaptation occurs at a level of visual processing that follows the integration of eye and head cues to gaze direction. Their participants were adapted on faces that evoke the Wollaston illusion, in which the direction that the face appears to look differs from its actual eye deviation due to the influence of head rotation on perceived gaze direction (Wollaston, 1824). They compared across sets of faces that were exactly matched in the lower-level features of the image, but appeared to be looking in different directions due to differences in the conjunction of head rotation and eye deviation. The changes in participants’ perception of gaze direction following adaptation were consistent with habituation having occurred to the perceived gaze direction of the Wollaston faces, where this is dependent on integration of eye deviation and head rotation, rather than to the actual deviation of the eyes. This indicates that adaptation operates within a higher-level, integrated representation of gaze direction, which relies on holistic processing of the face, rather than to specific features of the eye-region alone.

Similarly, adaptation to gaze direction can occur across a set of face images that differ substantially in their head rotation and eye deviation but maintain a constant direction of gaze relative to the viewer (Palmer and Clifford, 2017b). This suggests that the visual system codes the direction that other people are looking relative to ourselves as a higher-level or ‘abstract’ perceptual property, independent of the particular combination of head orientation and eye deviation that combine to signal a given direction of gaze in the current moment.

#### Is Gaze Adaptation Distinct From Other Directional Effects in High-Level Vision?

The direction of another person’s attention can be signaled to us by visual cues other than their eye deviation, such as their body rotation and pointing gestures. In addition, gaze direction can play a similar role to certain non-social cues, such as arrows, in directing the spatial focus of our own visual attention. Thus, there is a question of whether aftereffects following adaptation to gaze direction reflect changes in processing specific to gaze, or whether they are indicative of changes in processing in more general spatial or directional representations. The latter might include ‘social attention’ mechanisms that are agnostic to the particular cues that signal the direction of others’ attention (Cooney S. et al., 2015; Lawson and Calder, 2016), or even more generic (i.e., non-social) directional mechanisms. This question was partly addressed in the two original studies of gaze aftereffects. Jenkins et al. (2006) showed that gaze adaptation did not affect performance on a subsequent line bisection task, while Seyama and Nagayama (2006) found that adaptation to arrows did not induce gaze aftereffects. Subsequently, Bayliss et al. (2011) found that gaze adaptation had a direction-specific effect on how subsequently presented faces cued shifts in the subjects’ spatial attention, whereas adapting to a pointing stimulus did not. More recently, Palmer and Clifford (2017b) measured perceived gaze direction after adaptation to heads all turned to the same side over the range 0–50° but wearing dark glasses so that their eyes were not visible. They found that adaptation to turned heads did not induce significant gaze aftereffects, even though adaptation to turned heads does induce marked aftereffects in perceived head direction (Fang and He, 2005). Together these results indicate a degree of independence between representations of gaze direction and other directional or spatial cues.

While adaptation to gaze is specific from other types of directional stimuli, it generalizes from one face to another. For example, gaze adaptation does not show specificity for the sex of the face stimuli, such that there is no aftereffect contingent on the sex of the test following adaptation to a stimulus ensemble consisting of males with gaze averted 25° to one side and females 25° to the other (Kloth et al., 2015). Similarly, it is also common for studies of gaze adaptation to test the effects of adaptation on the perception of different identities to those adapted on (e.g., Palmer and Clifford, 2017a). Furthermore, gaze adaptation appears to be independent from identity processing in that it has been found to be unimpaired in patients with prosopagnosia (Duchaine et al., 2009).

### High-Level Influences on Gaze Adaptation

A small number of studies have examined how gaze aftereffects are influenced by higher-level aspects of the social context.

**FIGURE 2:**
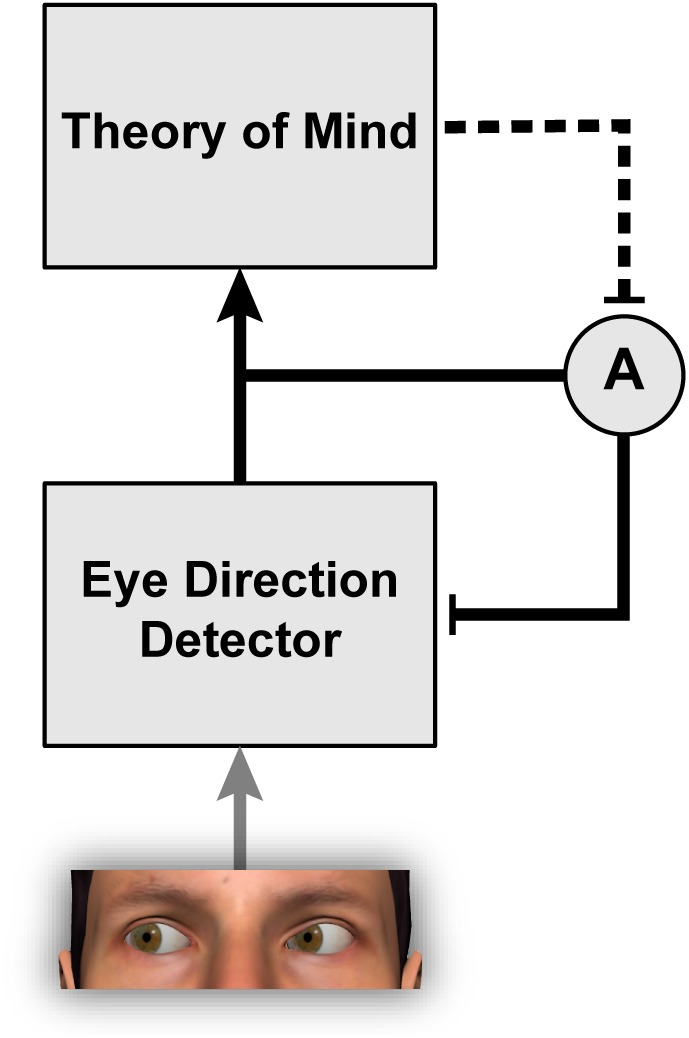
The phenomenon of gaze adaptation demonstrates that eye direction detection is a remarkably plastic process modifiable by the recent diet of stimulation. Eye direction information feeds into theory of mind by providing a cue about other people’s focus of attention, their knowledge, and their intentions (Baron-Cohen, 1995). The work of Teufel et al. (2009, 2013) indicates further that theory of mind is itself able to modulate the strength of adaptation A within the eye direction detector.

Interestingly, the strength of gaze adaptation appears to depend on the observer’s belief that the person used as the adapting stimulus can actually see (Teufel et al., 2009, 2013). The original study to report this effect (Teufel et al., 2009) involved an ingenious deception such that participants were led to believe that a pre-recorded video they were watching was actually a live camera feed from an adjacent room. In this video, participants saw a person gazing to the side while wearing a pair of goggles. In one condition, participants were led to believe that the goggles were transparent, and in another condition, that the goggles were opaque. Thus, participants believed that the adaptor could see in one condition but not the other, despite the visual features in the images being identical. The results revealed stronger gaze aftereffects when participants believed that the adaptor could see, suggesting that participants’ explicit beliefs about the person used as the adapting stimulus modulated the degree of adaptation (Figure 2). This result suggests that higher-level cognitive factors can determine whether the mechanisms that represent gaze direction are recruited to process a given visual stimulus or not. The interaction between theory of mind and social-perceptual mechanisms is discussed further in Teufel et al. (2010).

In their more recent study, Teufel et al. (2013) employed a similar set-up, except that this time participants were told that they were watching a pre-recorded video. In this experiment there was no significant difference in the magnitude of aftereffects between the two different types of adaptor, which the authors interpreted as indicating that mechanisms mediating theory of mind might be more strongly engaged when a stimulus video is actually believed to be a live link than when it is known to be a pre-recorded video.

The magnitude of gaze aftereffects has also been shown to be susceptible to modulation by the emotion of the adapting face, such that bigger aftereffects are evident following adaptation to happy compared to surprised faces (Seyama and Nagayama, 2006). This effect of emotion was evident despite the fact that Seyama and Nagayama were careful to use only information carried by the eyebrows and mouth to convey emotion while the eyes themselves were identical between the happy and surprised adapting stimuli. It is unclear by what mechanism emotional facial expression modulates the size of gaze aftereffects, but these results suggest, like those of Teufel and colleagues, that the engagement of adaptable representations of other people’s gaze direction (e.g., perhaps relating to the salience of the eye region) depends on the broader social context.

### Function of Gaze Aftereffects

Adaptation is generally held to offer functional benefits to the processing of sensory information. What benefits might adaptation confer to the processing of gaze direction?

In general, adaptation allows sensory systems to be self-calibrating in their mapping of aspects of the environment onto patterns of neural response and offers them the potential to optimize their coding properties to match the prevailing diet of stimulation (Clifford, 2005). Self-calibration encompasses recalibration and error-correction. Recalibration refers to changes in coding in response to changes in the environment. Error-correction is the process of adapting to changes in the system itself in an unchanging environment. Sensory systems, of course, have direct access neither to the state of the environment nor to their own internal state. However, they can remain self-calibrating by adapting their coding properties to keep the distribution of sensory response patterns constant (Benucci et al., 2013). In some aspects of visual processing, such as color vision, self-calibration allows the visual system to recalibrate to routine changes in the environmental illumination and thus achieve a high degree of constancy in the perception of the color of objects despite large changes in the wavelength distribution of light incident on the retina (Webster, 2015). In other visual modalities where strong adaptation is also observed, such as the processing of spatial orientation, the statistical distribution of environmental stimuli is typically less volatile and so the principal functional benefits of adaptation are in error-correction and optimization of coding efficiency. Given that the distribution of others’ gaze directions to which one is exposed is presumably fairly stable over time, it seems reasonable to assume that functional benefits of adaptation to gaze direction are also likely to be best understood in terms of error-correction and coding efficiency rather than recalibration to environmental changes. However, in the laboratory, artificially biasing the distribution of gaze directions to which an individual is exposed provides an excellent opportunity to measure the perceptual effects of gaze adaptation and, in turn, to make inferences about the underlying processes.

## What Gaze Aftereffects Reveal About the Sensory Coding of Gaze Direction

In the previous section, we saw that gaze aftereffects are indicative of a neural system that represents gaze direction as a property of the world abstracted from specific face features (e.g., combinations of head and eye direction), generalize across facial identities but are distinct from other high-level directional representations, and are recruited flexibly depending on the social context. In the current section, we examine how gaze aftereffects have substantiated a framework for understanding the sensory coding of gaze direction in the visual system, including the computational mechanisms that underlie these effects.

### Frame of Reference of the Adapted Representations

**FIGURE 3:**
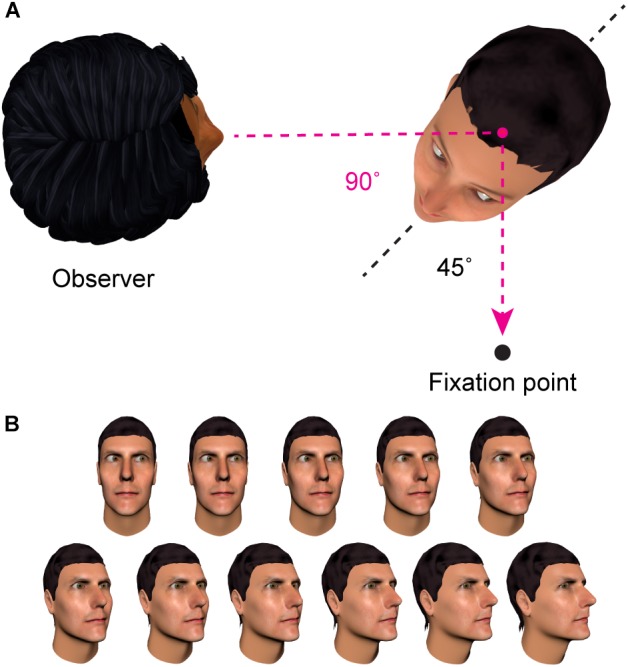
Contrasting frames of reference. **(A)** In this example, the individual on the right has gaze averted 90° relative to the observer (the ‘first person’ reference frame). In contrast, their eye direction relative to their own head is 45° (the ‘second person’ reference frame). **(B)** Across these face images, the direction of gaze is in the same rightwards direction relative to the viewer, but signaled by different combinations of head and eye angle. There is evidence that the brain contains representations of where other people are looking relative to oneself that are engaged regardless of the particular head and eye direction that combine to signal this direction of gaze in a given image (Palmer and Clifford, 2017b; Clifford, 2018; discussed in Section “Specificity of Gaze Adaptation”).

One characteristic of sensory coding that the adaptation paradigm can be used to probe is the *frame of reference* in which the nervous system represents information about other people’s direction of gaze. An important distinction can be drawn between a *first-person* reference frame, in which gaze direction is coded relative to the observer, and a *second-person* reference frame, in which gaze direction is coded relative to an axis of the stimulus (e.g., relative to the orientation of the head or body of the individual being observed). See Figure 3 for an illustration. Palmer and Clifford (2017b) adapted participants to a set of face images that maintained a particular direction of gaze in one reference frame, while varying the direction of gaze in the other reference frame. To test whether gaze adaptation involved representations in a first-person reference frame, they showed participants a series of face images that shared a particular direction of gaze relative to the observer (25° averted), but varied in their direction of gaze relative to the head of the stimulus. To test for gaze adaptation in a second-person reference frame, participants were shown stimuli that had eyes deviated 25° relative to the stimulus head, but where the direction of gaze relative to the viewer varied. The pattern of aftereffects they observed revealed adaptation specifically of first-person representations of gaze direction, with no evidence for adaptation in a second-person reference frame.

Whereas Palmer and Clifford (2017b) manipulated the yaw rotation of the head (i.e., around its vertical axis), Seyama (2006) investigated the effect of rotation in the image plane. Seyama found that adaptation to 35° horizontally averted gaze of a set of *upside-down* faces generated an aftereffect with *upright* test faces in a first-person reference frame. In other words, adaptation to upside-down faces gazing to their left (and therefore the observer’s left) produced a similar pattern of aftereffects as adaptation to upright faces gazing to their right (the observer’s left).

However, Seyama (2006) found that adaptation to faces rotated by 90° in the image plane also generated an aftereffect with upright test faces. Adaptation in a first-person reference frame should produce no aftereffect under these conditions, as the adapting stimuli were gazing upward or downward in the observer’s frame of reference whereas the test stimuli were gazing left or right. Instead, the results are consistent with adaptation in a second-person reference frame.

Seyama (2006) interpreted his results as evidence for adaptation occurring at both first- and second-person levels of representation. However, it is notable that in their study Palmer and Clifford (2017b) found no evidence for adaptation in a second-person reference frame and that the second-person aftereffects reported by Seyama (2006) were on average only around 40% of the magnitude of perceptual aftereffects reported in the original study of Seyama and Nagayama (2006). Thus, it appears that the adaptive coding of gaze direction in the human visual system probably occurs primarily, although not exclusively, in a first-person frame of reference.

### Using Adaptation to Reveal the Structure of the Neural Channels Coding Gaze Direction

Our perceptual experience contains a variety of information about the external world, and a fundamental question in neuroscience is how this information is represented or encoded in neural activity. This question can be considered both for lower-level perceptual properties, like the orientation of contours in our field of view, and more complex perceptual properties, like the gaze direction of a face. A strategy that appears to be employed across different levels of the visual hierarchy is *population coding*, whereby a perceptual property is represented in terms of the relative activity of a set of sensory neurons that are each tuned to different locations along the relevant stimulus dimension (Suzuki, 2005). For instance, area V1 contains neurons that are selective in their responding to edge-orientations, including neurons that respond most strongly to vertical edges, and neurons that respond most strongly to particular off-vertical edges (Hubel and Wiesel, 1968). In this way, the particular orientation of a presented stimulus can be encoded in terms of the relative firing rates that it elicits across this set of neurons.

The perceptual effects of adaptation can be understood in terms of how the pattern of activation elicited by a stimulus across a sensory population is modified by selective changes in the responsiveness of sensory neurons. In psychophysics, the concept of *sensory channels* refers to cell populations in the nervous system that display different tuning along a given stimulus dimension. Adaptation can be modeled as a reduction in the responsiveness of a set of sensory channels proportional to how strongly each channel is engaged by the adapting stimulus (Graham, 1989). In this way, a given test stimulus will produce a different pattern of activation across sensory channels before adaptation compared to after adaptation, due to uneven changes in the sensitivity of the channels. The perceptual effects of adaptation reflect how altered channel sensitivities affect the ‘end result’ of population coding, so can be used to probe how a stimulus property is represented across a system of channels. In the context of gaze perception, single-cell recording studies in macaque monkeys have identified cells in the temporal cortex (specifically, the anterior STS) that not only respond selectively to faces but also respond differentially to the gaze direction of the face (Perrett et al., 1985; De Souza et al., 2005). In humans, haemodynamic responses in anterior STS similarly indicate the existence of distinct cell populations tuned to different gaze directions (Calder et al., 2007; Carlin et al., 2011), and various sub-cortical areas are also implicated in the rapid detection of direct eye contact (Senju and Johnson, 2009). Studies that investigate the perceptual effects of adaptation to gaze direction have built upon these findings by providing important new insights into how gaze direction may be encoded across a set of gaze-selective sensory channels (Clifford, 2018).

One functional characteristic of gaze processing of which aftereffects can be diagnostic is the *number of sensory channels* involved in representing gaze direction. The earliest studies showed dissociable perceptual aftereffects of adaptation to leftward and rightward averted gaze (Jenkins et al., 2006; Seyama and Nagayama, 2006) indicating that there are dissociable neural mechanisms coding these directions, likely located in the right anterior STS of the human brain (Calder et al., 2007). The relative activation of *two opponent channels* tuned to leftward and rightward gaze could in principle code the full range of horizontal gaze directions that we encounter, with direct gaze represented by equal activation of these channels and increasingly averted gaze represented by increased activation in one channel over the other. However, psychophysical adaptation studies further suggest the existence of a channel coding explicitly for gaze directed at the observer, pointing to the existence of at least three sensory channels coding for horizontal gaze direction (Calder et al., 2008; Palmer and Clifford, 2017a).

Calder et al. (2008) investigated the range of test gaze directions that observers categorized as being directed at them, and observed opposite effects of adaptation on perceived gaze direction depending on whether adaptation was to a series of faces (i) all gazing directly at the observer or (ii) alternating between leftward and rightward gaze averted by 25°. Calder et al. (2008) reasoned that, within each of these two adaptation conditions, channels tuned to leftward and rightward gaze should be engaged to the same extent. Consequently, in a two-channel opponent system, the two adaptation conditions should have qualitatively the same effect on the range of test gaze directions categorized as direct. However, adaptation to direct gaze was found to narrow the range of test gaze directions categorized as direct whereas adaptation to alternating leftward and rightward gaze broadened the range (see Lawson et al., 2009, 2011 for analogous findings regarding adaptation to body and head direction, respectively). Calder *et al.* reasoned that the most parsimonious account of this pattern of data was a system of three broadly tuned channels tuned to leftward, rightward and direct gaze, respectively. Under such a system, adaptation to direct gaze would engage primarily the direct channel, causing the range of subsequent test directions perceived as direct to narrow. Conversely, adaptation to alternating leftward and rightwards gaze would preferentially engage the leftward and rightward channels, causing the range of gaze directions perceived as direct to broaden.

The use of a categorical measure of perceived gaze direction (e.g., left/direct/right) requires observers to adopt a decision criterion as to where precisely the boundaries between categories lie. It is conceivable that adaptation might affect such subjective category boundaries in a systematic way, rather than the perceptual experience of gaze direction *per se* (Storrs, 2015). For example, if exposure to a series of directly gazing faces served as a repeated reference as to what constitutes direct gaze then it might both narrow the range of test faces categorized as direct and make the location of the subjective category boundaries less variable on a trial-by-trial basis. If this were the case, then one might in principle be able to account for the pattern of data reported by Calder et al. (2008) within the framework of a two-channel opponent system. In other words, adaptation to direct gaze might not affect *perception* of gaze direction, but rather how a given perceived direction of gaze is categorized.

Palmer and Clifford (2017a) revisited the question of what channel structure underlies the coding of horizontal gaze direction using a different response method. Participants were required to use a pointer to indicate perceived direction, avoiding the need for them to adopt subjective category boundaries in their responding. Although not finding clear evidence of an aftereffect of adaptation to direct gaze (see also Kloth and Schweinberger, 2010), Palmer and Clifford observed in their data a novel characteristic diagnostic of the existence of a direct channel. Specifically, they found that the magnitude of aftereffects to 25° averted gaze was tuned for test direction, with the maximum aftereffects evident for test stimuli averted 10–15° to the same side as the adaptor. This finding was replicated in Palmer and Clifford (2017b) and Palmer et al. (2018).

**FIGURE 4:**
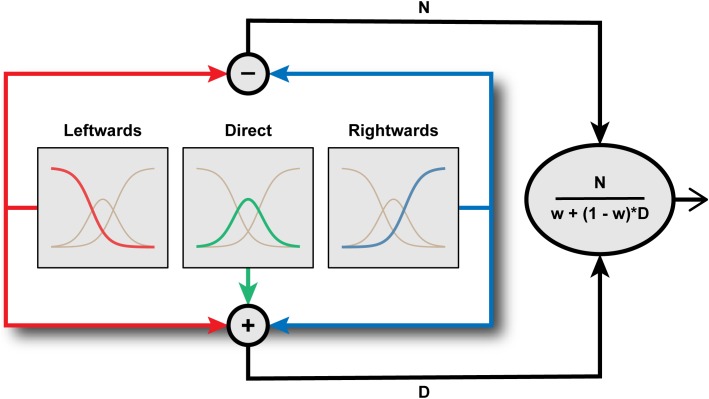
Inside the Eye Direction Detector. Schematic representation of the functional architecture proposed by Palmer and Clifford (2017a) to underlie the coding of horizontal gaze direction. Gaze direction is encoded by the pattern of activation across three channels tuned to leftward, direct, and rightward, respectively. The outputs of these channels are then combined through a process of divisive normalization of an opponent left-right signal to generate a metric estimate of gaze direction.

Using computational modeling to simulate hypothetical channel structures, Palmer and Clifford (2017a) demonstrated that the tuning of aftereffect magnitude for test direction is characteristic of a system comprising a small number of broadly tuned mechanisms whose activity is subject to divisive normalization (Figure 4). Specifically, leftwards and rightwards channels are combined in opponent fashion, as in a simple opponent model, but this opponent signal is divided by the sum of the signals across all (leftward, rightward, and direct) channels. Their simulations supported the intuitive notion that the effects of adaptation on perception should be most evident when the test stimulus engages channels differentially affected by the adapting stimulus. This leads to distinct predictions for the tuning of aftereffects in two-channel opponent and three-channel systems. For example, following adaptation to leftward averted gaze, a system of only two opponent channels would produce the strongest aftereffects for direct test stimuli, as this is the direction for which the strongly adapted (leftwards) and relatively unadapted (rightward) channels are equally engaged. In a three-channel system, however, the strongest aftereffects following adaptation to leftwards averted gaze would be evident for a test direction where leftwards and direct channels are equally engaged, i.e., moderately averted gaze to the same side as the adaptor, as observed empirically. The findings of Palmer and Clifford (2017a) thus support the conclusions of Calder et al. (2008) that the coding of horizontal gaze direction is inconsistent with the operation of a two-channel opponent system but can be parsimoniously accounted for within a three-channel framework.

It is interesting to note the architectural similarity between the model of gaze processing proposed by Palmer and Clifford (2017a) and the channel structure of the early stages of the color vision pathway (Gegenfurtner and Sharpe, 1999). Normal human color vision is subserved by three classes of retinal cone photoreceptor with overlapping bands of wavelength selectivity. Signals from these three chromatic channels are combined in opponent fashion in the sub-cortical visual pathway and subject to normalization (Solomon and Lennie, 2005). Palmer and Clifford’s computational modeling similarly highlights the roles of channel opponency and divisive normalization in the coding of gaze direction. Divisive normalization is a form of gain control, ensuring that the relative activation across channels provides a code that is robust to variation in the absolute level of stimulation. In the present context, the encoded gaze direction is normalized to the pooled activity across gaze-selective sensory channels. This ensures that the encoded gaze direction is not affected by extraneous variables that might influence activity across these channels (e.g., stimulus contrast), but rather relates only to the proportional difference in activity between gaze-selective channels. Divisive normalization has been argued to be a canonical feature of nervous system function (Carandini and Heeger, 2012), though it is relatively unexplored in the context of higher-level, social vision. In general, the effect of adaptation on sensory coding is complicated by its potential to act on both driving and suppressive mechanisms (Solomon and Kohn, 2014). Here, the precise tuning profile of gaze aftereffect magnitude generated by the model to fit the empirical data arises because signals from the adapted channel(s) feed into not only the driving mechanism (‘N’ in Figure 4) but also into the normalization signal (‘D’ in Figure 4).

In summary, attempts to account for the specific characteristics of gaze aftereffects (e.g., their tuning across test directions) has substantiated a computational framework for understanding the sensory coding of gaze direction in the visual system.

### Gaze Adaptation in People With Autism Spectrum Disorder

Autism spectrum disorder is a heterogeneous developmental condition whose characteristics include atypicalities in social communication and interaction (American Psychiatric Association, 2013), including gaze-based behaviors (Baron-Cohen, 1995). To understand the social mind in ASD, it is important to study both the perceptual mechanisms that furnish the individual with information about other people (e.g., the eye direction detector; Figure 2) as well as more cognitive processes that employ or interpret this information within the broader social context (e.g., theory of mind). There is now a significant body of research investigating perceptual function in ASD (e.g., Simmons et al., 2009; Marco et al., 2011), and many theories emphasize how systematic differences in the processing of sensory information may contribute to diverse features of this condition (e.g., Happé and Frith, 2006; Mottron et al., 2006; Pellicano and Burr, 2012; Rosenberg et al., 2015; Palmer et al., 2017). However, the sensory mechanisms that underlie perceived gaze direction are yet to be comprehensively examined in ASD, despite the relevance of gaze perception to many social-cognitive functions. As we have seen, perceptual adaptation is a useful tool for probing how information about other people’s direction of gaze is flexibly encoded in the visual system.

**FIGURE 5:**
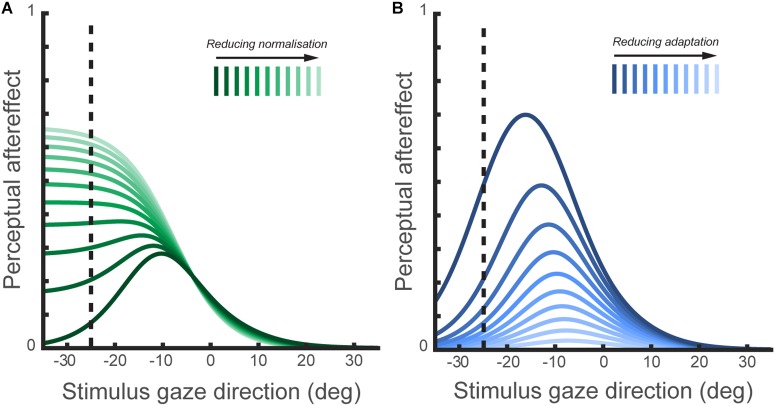
**(A)** The simulated effect of *normalization mechanisms* on the tuning of perceptual aftereffects. This figure shows the size of perceptual aftereffects predicted by the model of perceived gaze direction (illustrated in Figure 4) following adaptation to 25° leftward gaze, across a range of test stimulus gaze directions. In the model of perceived gaze direction, the encoded gaze direction is normalized to the summed activation across gaze-selective sensory channels. The plotted lines show the simulated aftereffects for a series of models ranging from ‘full’ normalization of the encoded gaze direction to a complete lack of normalization. As the degree of normalization is reduced, the tuning of aftereffects across test gaze directions change in a systematic way. Thus, perceptual aftereffects observed following adaptation to gaze direction may be indicative of differences between individuals or groups in the operation of normalization mechanisms in the coding of gaze direction. **(B)** The simulated effects of *channel adaptability* on perceptual aftereffects. The plotted lines show the simulated aftereffects for a series of models with the same degree of normalization, but where exposure to the adapting stimulus results in either a stronger or weaker change in subsequent channel sensitivities. The degree of channel adaptability scales the magnitude of perceptual aftereffects, but has a less distinct effect on the tuning of aftereffects across stimulus gaze directions compared to the effect of varying normalization shown in **(A)**.

Reduced effects of adaptation to gaze direction have been observed in ASD both in children (Pellicano et al., 2013) and adults (Lawson et al., 2017). Palmer et al. (2018) also recorded perceptual aftereffects in a sample of adults with ASD, and fitted a computational model of the sensory coding of gaze direction to this data to characterize the function of specific mechanisms involved in gaze processing. This included simulating the effect of varying either the degree of divisive normalization (‘1-w’ in Figure 4) or the degree of channel adaptability in the model of horizontal gaze coding proposed by Palmer and Clifford (2017a). A recent hypothesis is that altered divisive normalization processes may contribute to a wide array of the behavioral consequences in ASD (Rosenberg et al., 2015), though it had not been explored whether any differences in divisive normalization computations were apparent in the context of perceived gaze direction. On the basis of their computational modeling, Palmer et al. (2018) predicted that a reduction in the degree of divisive normalization in the gaze system should lead to a broader tuning profile of gaze aftereffect magnitude as a function of test direction (Figure 5). In contrast, reduced sensitivity to the recent history of sensory stimulation more generally (i.e., reduced adaptability of sensory channels) would lead to a reduction in the overall magnitude of perceptual aftereffects, rather than a change in the tuning profile as a function of test direction.

However, 27 adults with a diagnosis of ASD showed no difference from matched neurotypical controls in either the overall magnitude of their gaze aftereffects or the degree of divisive normalization inferred from fitting the model to their data. On the basis of a Bayesian statistical analysis, Palmer et al. (2018) concluded that their results provide strong support for there being no difference between ASD and control groups in how the effects of adaptation differ across test directions. Nor was there a significant correlation between the strength of adaptation or normalization at an individual level and autistic features (ADOS and AQ scores). As described in the previous section, the perceptual effects of adaptation to averted gaze can be indicative of several functional mechanisms, including (i) the flexible adjustment of channel gain in response to the recent history of sensory stimulation, (ii) the divisive normalization of sensory responses, and (iii) the channel structure coding for horizontal gaze direction. Thus, the robust magnitude and profile of perceptual aftereffects observed in this study is a testament to the typical coding of other people’s gaze direction in the visual system in adults with ASD, despite the social-cognitive differences that are characteristic of this condition.

The observation by Palmer et al. (2018) of strong gaze aftereffects in adults with ASD appears at odds with previous findings of reduced effects of adaptation to gaze direction in ASD both in children (Pellicano et al., 2013) and adults (Lawson et al., 2017). A methodological difference between these studies is the method used to quantify shifts in perceived gaze direction associated with adaptation. In Palmer et al. (2018) participants indicated the perceived direction of gaze metrically, by setting the rotation of a pointer, while in the previous two studies participants made a categorical judgment as to whether the face was looking directly toward them or not. The difference between studies may therefore reflect a difference between groups in how gaze directions are categorized. For instance, when using categorical measures of gaze perception, a given shift in perceived gaze direction following adaptation will be most apparent when it occurs *across* category boundaries (e.g., perceived gaze direction shifting from an angle of gaze consistently categorized as ‘averted’ to one consistently categorized as ‘direct’). The effects of adaptation may thus be less distinct in individuals who classify a wider range of gaze deviations as direct, or who have a less sharp transition between what they judge as being ‘direct’ and ‘averted’ gaze. In the two earlier studies of gaze adaptation in ASD (Pellicano et al., 2013; Lawson et al., 2017), participants with ASD more commonly categorized gaze as direct at baseline compared to neurotypical controls. Thus it might be that the reduced effects of adaptation reported in these studies reflect a greater difficulty in detecting adaptation effects in the ASD group when using categorical measures, rather than a difference in the effects of adaptation on the coding of gaze direction *per se*.

## Conclusion and Future Directions

In this review, we have discussed the marked changes in perception of gaze direction that occur following adaptation to faces with a particular direction of gaze (Jenkins et al., 2006; Seyama and Nagayama, 2006). These aftereffects can be measured robustly and are evident across faces with a range of different test gaze directions (Palmer and Clifford, 2017a,b; Palmer et al., 2018). There is psychophysical evidence that gaze aftereffects reflect habituation of neurons that are specifically involved in the representation of other people’s gaze direction, rather than merely being inherited from changes in processing at lower levels of the cortical visual hierarchy or reflecting habituation at the level of more generic directional representations (Jenkins et al., 2006; Stein et al., 2012). Moreover, gaze aftereffects are indicative of a level of visual processing in which different facial features are integrated to produce our sense of where others look (e.g., head and eye direction; Stiel et al., 2014; Palmer and Clifford, 2017b; Clifford, 2018). Adaptation to gaze direction is associated with changes in the neural processing of faces that are detectable in both haemodynamic responses (Calder et al., 2007) and scalp potentials (Schweinberger et al., 2007; Kloth and Schweinberger, 2010). The effects of adaptation on cortical processing have been observed in the right anterior STS and right IPL (Calder et al., 2007), consistent with a view of gaze-specific processing emerging in higher-level visual pathways in temporal cortex (Carlin and Calder, 2013). However, the contribution of sub-cortical areas of the ‘social brain’ to gaze aftereffects, such as the superior colliculus, amygdala, and pulvinar (Senju and Johnson, 2009), is currently unknown.

Exploring the determinants of gaze aftereffects has provided insights into how other people’s direction of gaze is encoded in the visual system, and the functional and computational mechanisms upon which this process depends (Calder et al., 2008; Palmer and Clifford, 2017a). This work has focused so far on the representation of horizontal gaze direction acroive sensory channels. Gaze is a multi-dimensional phenomenon, however, such that the focus of another person’s gaze can be described in spherical coordinates relative to their face, with both polar and azimuthal angles as well as a particular depth of fixation. Further work thus remains to investigate the effects of adaptation on the perception of gaze deviations along the vertical and oblique axes (Cheleski et al., 2013). This is an important extension, not only because the geometry of the eyes and head is very different along the horizontal and vertical directions, but also because of the different social signals conveyed by horizontally and vertically averted gaze. For example, downwards gaze can signal shame or embarrassment (Darwin, 1965), and gaze can be averted downwards while still being directed at the viewer (Lawson et al., 2011). Similarly, there is a question of how adaptable mechanisms that carry information about the depth of fixation (i.e., gaze convergence: Stiel et al., 2014) combine with mechanisms that represent gaze direction, to jointly specify the focus of other people’s gaze in three-dimensional space (Nguyen et al., 2018). In addition, the *positional specificity* of gaze aftereffects (e.g., retinotopic or spatiotopic), and by implication the spatial receptive field properties of the mechanisms that represent gaze direction, are currently unknown.

Perceptual aftereffects are also apparent for cues to the direction of other people’s social attention other than the eyes, including heads (Fang and He, 2005), static and walking bodies (Lawson et al., 2009; Benton et al., 2016), and pointing hands (Cooney S.M. et al., 2015). There is some evidence for a degree of cross-adaptation between different directional cues, namely from head to body direction but not vice versa (Cooney S. et al., 2015), which may indicate overlap in the mechanisms representing other people’s direction of social attention derived from different bodily features. However, Lawson and Calder (2016) found no significant cross-adaptation either way between head and body direction. Even if the mechanisms prove to be distinct, further research should determine whether or not the channel structures and functional processes underlying the coding of gaze direction and other indicators of social attention share similar principles of organization. For example, while the narrowband model of head viewpoint coding proposed by Chen et al. (2010) contrasts with the broadband model of perceived gaze direction implemented in Palmer and Clifford (2017a), a broadband model might also be able to account for the effects of adaptation on the perception of head and body direction (Lawson et al., 2009, 2011).

Finally, the perceptual adaptation paradigm is useful as a method for assessing plasticity and the sensory coding of gaze direction in conditions with apparent differences in the response to other people’s eye gaze, such as autism (Pellicano et al., 2013; Lawson et al., 2017; Palmer et al., 2018), schizophrenia (Tso et al., 2012), and social anxiety disorder (Jun et al., 2013). Understanding the function of basic sensory mechanisms in gaze perception may form an important complement to research on higher-level social cognition in these conditions (Baron-Cohen, 1995; Green et al., 2015).

## Author Contributions

CC wrote the initial draft of the manuscript. CP made extensive revisions to the original draft and prepared all of the figures. CC and CP approved the final version of the manuscript.

## Conflict of Interest Statement

The authors declare that the research was conducted in the absence of any commercial or financial relationships that could be construed as a potential conflict of interest.
